# Disordered Eating among Preadolescent Boys and Girls: The Relationship with Child and Maternal Variables

**DOI:** 10.3390/nu4040273

**Published:** 2012-04-17

**Authors:** Sónia Gonçalves, Margarida Silva, A. Rui Gomes, Paulo P. P. Machado

**Affiliations:** Department of Applied Psychology, School of Psychology, University of Minho, Campus de Gualtar, Braga 4710, Portugal; Email: a49038@alunos.uminho.pt (M.S.); rgomes@psi.uminho.pt (A.R.G.); pmachado@psi.uminho.pt (P.M.)

**Keywords:** eating behavior, body dissatisfaction, gender differences

## Abstract

Objective: (i) To analyze the eating behaviors and body satisfaction of boys and girls and to examine their mothers’ perceptions of these two domains; and (ii) to evaluate eating problem predictors using child body mass index (BMI), self-esteem, and body satisfaction as well as maternal BMI, eating problems, and satisfaction with their child’s body. The participants included 111 children (54.1% girls aged between 9 and 12 years old) and their mothers. Assessment measures included the *Child Eating Attitude Test*, the *Self-Perception Profile for Children*, the *Eating Disorders Questionnaire*, and the *Child Eating Behavior Questionnaire*. Child and maternal measures also included BMI and *Collins Figure Drawings*. Results: (i) No association between child and maternal BMI for either sex was found; (ii) no difference was found between boys and girls with regard to eating behavior; (iii) most children revealed a preference for an ideal body image over their actual body image; (iv) most mothers preferred thinner bodies for their children; (v) greater BMI was related to higher body dissatisfaction; and (vi) child BMI and dissatisfaction with body image predicted eating disturbances in boys, whereas self-esteem, maternal BMI, and eating behavior predicted them in girls. Discussion: Maternal eating problems and BMI were related to female eating problems only.

## 1. Introduction

Eating disorders among boys and girls are rare, but many children demonstrate behavioral eating disturbances [1]. Behaviors and attitudes such as restrained eating, fear of being fat, distorted body image, binge eating, and purging are common among preadolescents of both sexes. Specifically, 50% of the children wanted to weigh less than their current weight and 16% had attempted to lose weight [2]. Other estimates of dieting to lose weight varied from 20% to 55.6% for girls and 31% to 39% for boys, whereas 43.5% of girls and 43.5% of boys reported exercising to lose weight [3].

Despite the interest in these findings, child-feeding practices and eating problems in childhood are not well understood. In addition, the diagnostic groups for feeding and eating disorders in childhood have numerous problems, including the wide and inconsistent use of terminology as well as limited research [4]. Moreover, much of the available research on eating disturbances and body image in children has focused on girls [5] and on maternal eating behaviors, feeding practices, and on the effects these behaviors have on young girls [6,7,8]. Few studies have compared boys with girls regarding eating behaviors and the role of personal and maternal variables in predicting eating problems. 

As such, this study had two main goals. The first goal was to analyze the eating behaviors and body satisfaction of boys and girls and to analyze their mothers’ perceptions on these two domains. Thus, this study compared the eating behaviors of boys with girls aged 9–12 years old, and the maternal perceptions of their children’s eating behaviors. This study also compared the body satisfaction of boys with that of girls as well as maternal body satisfaction with that of their child. This goal was based on previous suggestions [9,10] that considered the investigation of the meaning of gender in body image and eating problem research to be important. Before testing these differences in body image and eating problems according to gender, we evaluated a possible association between maternal and child body mass index (BMI) for both sexes because some studies [11] have found that child BMI is significantly correlated with parental BMI (although there is a closer association with maternal than with paternal BMI). 

The second goal of this study was to evaluate the predictors of eating problems using child BMI, self-esteem, body satisfaction and maternal BMI, eating problems, and satisfaction with the body of their child. These latter variables of mothers have been shown to predict disordered eating in young children. For example, recent research [12,13] has focused on the role of the family in patterns of eating behaviors and the impact of BMI on the eating behaviors and body image of children. The assumptions that eating patterns are inherited and that this genetic contribution operates in a particular environmental context have fueled this type of research [14]. For example, while a predisposition to obesity might be due largely to individual differences in metabolism, it is also possible that behavioral and environmental pathways are significant. On the other hand, messages concerning dieting and thinness are widespread in the environment, and the primary place where this occurs is the family, where children see dieting behaviors in action [2]. Considering these findings, this study evaluated maternal perceptions of their children’s body images and eating behaviors. We assumed that the family is the primary social context and that mothers play an important role in the eating behaviors of young children [15].

In addition to analyzing eating behaviors from the perspectives of children and their mothers, this study also included measures of body image, body satisfaction, and self-esteem. These variables were chosen because they are related to eating disturbances in children and adolescents [10]. According to previous research, by the age of 8 to 13 some children already believe it would be better to weigh less due to their body image, although they do not necessarily attempt to lose weight [2]. In fact, two longitudinal studies [16,17] found that negative feelings toward body image predicted eating problems and eating disorder symptoms. According to Stice and Shaw [18] there is consistent support for the assertion that body dissatisfaction is a risk factor for eating pathology. We included self-esteem as a predictor of eating problems because research has demonstrated that body image disturbances and eating problems in children are frequently related to low self-esteem [3,5]. 

With regard to the main goals of this study, we examined six specific research questions:

(i) Is there an association between maternal and child BMI for both sexes?
(ii) Are there differences between boys and girls with regard to eating behaviors?
(iii) Are there differences between mothers of boys and girls with regard to perceptions of child’s eating behaviors?
(iv) Are there differences between boys and girls with regard to body dissatisfaction?
(v) Are there differences in maternal perspectives of body dissatisfaction for mothers of boys *versus* girls?
(vi) What variables predict eating problems in girls and boys after controlling for the personal and psychological variables of children and mothers?

## 2. Method

### 2.1. Participants

A convenience sample of 111 children and their respective mothers (*n* = 111) was evaluated. To ensure socioeconomic diversity, all participants attended public schools.

The children included 51 boys (45.9%) and 60 girls (54.1%) aged between 9 and 12 years (M = 10.08; SD = 0.81). The mean maternal age was 39.58 years (SD = 5.27). Most mothers were married (89.2%). All mothers had received at least an elementary education (25.2% of mothers had 9 years of education, 25.2% had 12 years of education, and 15.3% had college education).

### 2.2. Measures

Demographic and anthropometric information. The demographic information (e.g., gender and age) and anthropometric measures (e.g., height and weight to calculate BMI) of mothers and children were collected. The prevalence of underweight, normal weight, overweight, and obese participants was calculated [19] to correspond with traditional adult cut-offs as well as specific values for children considering sex and age.

#### 2.2.1. Child Measures

Children’s Eating Attitude Test (ChEAT) [20,21]. The child version of the Eating Attitude Test (EAT) is a 26-item, 6-point Likert scale ranging from *always* to *never*. High test-retest and internal reliability coefficients have been found for both girls and boys [20,22]. In addition, the ChEAT factor structure for young girls [23] closely corresponds to the EAT factor structure in adolescent girls and women [24,25]. A score of 20 or above suggests the presence of an eating disorder, although the test cannot provide a definitive diagnosis [20]. The Portuguese version of the EAT has three dimensions: (i) diet (α = 0.76); (ii) bulimia and preoccupation with food (α = 0.50); and (iii) food control (α = 0.50). Cronbach’s alpha was 0.72 for the ChEAT.

Collins’ Figure Drawings (CFD) [26]. The CFD is a pictorial instrument consisting of seven preadolescent body figures ranging from very thin (scored 1) to obese (scored 7). This instrument is the child version of body drawings for adults [27]. Seven male and female figures of children were created to illustrate body weights ranging from *thin* to *obese*. Participants used the CFD same-gender child figure to make self (*Which picture looks the most like you*?) and ideal-self selections (*Which picture shows the way you want to look*?). The CFD score was determined by subtracting the value of the self-selection from the value of the ideal-self-selection. Negative scores indicate desires for thinner body shapes, whereas positive scores indicate desires for heavier body shapes. Collins (1991) reports an overall 3-day test-retest coefficient for current self of 0.71. The correlation between pictorial self-selection and weight was 0.36 while the correlation between BMI and self-rating was 0.37.

Self-Perception Profile for Children (SPPC) [28,29]. The SPPC is a 36 item self-report questionnaire that assesses child self-esteem. This scale provides a total score and five subscales representing five domains of self-esteem: (i) scholastic competence; (ii) social acceptance; (iii) athletic competence; (iv) physical appearance; (v) behavioral conduct, as well as global self-esteem. In this study, only the global self-esteem (α = 0.73) was considered for the analysis. Each item contains the following opposite descriptions: *Some children often forget what they have learned* but *other children are able to remember things easily*. Children must choose the description that fits them and then indicate the degree of fit (true or very true) and then indicate whether the description is somewhat true or very true for them. Accordingly, each item is scored on a 4-point Likert scale in which higher scores reflect more positive self-views. A total score was computed for the self-esteem domain and the global self-esteem scale by adding the items’ values. This sum was then divided by the total number of items to form a subscale. 

#### 2.2.2. Maternal Measures

Eating Disorder Examination Questionnaire (EDE-Q) [30,31]. The EDE-Q is a self-report version of the *Eating Disorder Examination *[32], a well-established, investigator-based interview. This version contains 28 items answered using a 7-point Likert-scale that ranges from 0 (*none*) to 6 (*every day*). The EDE-Q includes four subscales that measure the severity of aspects related to pathological eating behaviors that have occurred in the past 28 days, These subscales include: (i) restraint (α = 0.74); (ii) eating concern (α = 0.74); (iii) shape concern (α = 0.86); and (iv) weight concern (α = 0.75). Greater scores in each dimension indicate a higher likelihood of disordered eating behaviors. A global score was calculated from the average of the four subscale scores (α = 0.92). 

Child Eating Behavior Questionnaire (CEBQ) [33,34]. The CEBQ is a multi-dimensional, parent-report survey that measures child eating behavior using a 5 point Likert scale (from 1 = *never *to 5 = *always*). This measure examines individual differences in eating style that are hypothesized to contribute to below or above average participant weights. The scale constructs were derived from the literature on eating behavior and weight. The CEBQ contains eight subscales: (i) satiety response (α = 0.79); (ii) eating speed (α = 0.88); (iii) selectivity (α = 0.73); (iv) emotional undereating (α = 0.70); (v) food responsiveness (α = 0.82); (vi) enjoyment of food (α = 0.89); (vii) emotional overeating (α = 0.77); and (viii) desire to drink (α = 0.82). 

Collins figure drawings (CFD) [26]. In this study, mothers used the pictorial instrument described above to make two selections: (i) *Which picture looks the most like your child look*? (same-gender child figure); and (ii) *Which picture shows the way you want your child to look*? (same-gender child figure). The score was determined by subtracting the value for the current figure from the value for the desired figure. Negative scores indicate a desire for a thinner body shape, while positive scores indicate a desire for a heavier body shape. 

### 2.3. Procedure

This study was reviewed and approved by the internal review board of Research Center of Psychology (University of Minho) in October 2010, and conformed to both National and European regulations on conducting research with human participants and on the management of personal data. Data collection involved the solicitation of permission from the Portuguese Ministry of Education and regional school authorities; the solicitation of permission from school directors to collect data for the research project; and finally the discussion of data collection with teachers and students at school visits. This research was presented as a study of mother and child eating behaviors. No exclusion criteria were considered. The questionnaires were provided to children during class time by one of the authors who read the instructions to each class. The CEBQ, CFD, and EDE-Q were sent to the mothers of selected children via teachers. Parents provided written consent for their children and themselves prior to data collection. All participants were informed that their involvement was voluntary and anonymous, and all the questionnaires delivered were returned. 

Body satisfaction was evaluated using the CFD for children. The score was determined by subtracting the value of the self-selection from the value for the ideal-self-selection. Negative scores indicate desires for thinner body shapes, whereas positive scores indicate desires for a heavier body shapes. Values equal to zero indicate no body dissatisfaction. Maternal satisfaction with their child’s body was evaluated using the CFD for children. Its score was determined by subtracting the value of the child selection from the value of the ideal selection. Negative scores indicate maternal desires for their child to have a thinner body shape, whereas positive scores indicate maternal desires for them to have a heavier body shape. Values equal to zero indicate no body dissatisfaction.

### 2.4. Data Analyses

First, an exploratory data analysis tested parametric test assumptions. We did not find assumption violations in most cases. In cases in which normality was not assumed, we computed parametric test statistics and their non-parametric equivalent [35]. The results from both tests allowed us to make the same general conclusions. As such, we present only the parametric test results because they are more robust and allow us to use multivariate analyses, thereby reducing the number of tests conducted and the probability of a Type I error.

To evaluate the associations between nominal variables, chi-square tests were conducted. To evaluate between-group differences with regard to BMI, independent-samples *t*-tests were conducted. To evaluate between-group differences with regard to ChEAT and CEBQ scores, we applied multivariate analyses of variance. To predict disordered eating behavior in both groups of children, we applied a regression analysis with a blocked entry procedure. Considering the distribution of the children’s BMI, this variable was dichotomized in order to allow comparison between children with normal weight *versus* overweight/obese, with the exception in the prediction model were BMI entered as a continuous variable. The same procedure was used regarding maternal BMI for both analyses. In the same way, the children’s body dissatisfaction and maternal reports of child body satisfaction were dichotomized (body satisfaction *versus* body dissatisfaction). 

## 3. Results

### 3.1. Child and Maternal BMI

The BMI percentile was adjusted for age and gender, and 38 boys were of normal weight (74.5%), and 13 (25.5%) were overweight/obese. Furthermore, 43 girls were of normal weight (71.7%), and 17 (28.3%) were overweight/obese. No underweight children were found. Approximately 33% (*n *= 36) of mothers were overweight (25 ≤ BMI < 30) and approximately 11% (*n *= 12) were obese (BMI ≥ 30).

Child and maternal BMIs were analyzed to explore the association between child BMI and sex as well as the association between maternal BMI and child BMI. We did not find a relationship between child BMI percentile and sex (χ²_(1)_ = 0.11, *p* = 0.83) or between maternal BMI and child BMI (boys χ²_(2)_ = 2.42, *p* = 0.30; girls χ²_(3)_ = 4.65, *p* = 0.20). No differences were found between girls and boys with regard to maternal BMI (boys M = 24.74; SD = 3.90 *versus* girls M = 24.84; SD = 4.30; *t*(109) = −0.13, *p* = 0.90).

### 3.2. Eating Behaviors in Boys and Girls

Child eating behaviors were analyzed to explore between-sex differences. The distributions of the ChEAT total scores were similar for both genders (boys M = 9.02; SD = 7.15; girls M = 7.72; SD = 6.52; *t*(109) = 1.00, *p* = 0.24). No between-group differences were found with regard to the eating behavior subscales (Wilks’ λ = 0.97, *F*(3, 107) = 0.94, *p* = 0.43, η^2^= 0.03). Maloney *et al*. [20] argued that ChEAT scores of 20 or higher should be regarded as indicating risk for an eating disorder; 9.8% (*n* = 5) of the boys and 8.3% (*n* = 5) of the girls scored above this threshold. No associations were found between sex and ChEAT scores (χ²_(1)_ = 0.07, *p* = 0.52).

### 3.3. Maternal Perceptions of Child Eating Behaviors

Maternal perceptions of child eating behaviors were analyzed to explore the differences between mothers of boys and those of girls. To do so, maternal CEBQ scores were analyzed. These results did not reveal between-group differences with regard to the CEBQ subscales (Wilks’ λ = 0.95, *F*(8, 102) = 0.68, *p* = 0.62, η^2^= 0.06). 

### 3.4. Body Satisfaction in Boys and Girls and Maternal Reports of Child Body Satisfaction

Approximately 55% of the boys and 58.3% of the girls reported body dissatisfaction. Of these children, 78.6% of the boys and 80% of the girls reported desires for thinner bodies. Table 1 presents the percentages of body image satisfaction in boys and girls. There was no significant association (χ²_(2)_ = 0.15, *p* = 0.93) between sex and body satisfaction. Approximately 55% of the boys’ mothers and 43.3% of the girls’ mothers were not satisfied with their child’s body image. Of these mothers, 57.1% of the former group and 61.5% of the latter group desired their child to have a thinner body. Table 1 presents the percentages of the maternal satisfaction with their child’s bodies. No association (χ²_(2)_ = 1.6, *p* = 0.45) was found between maternal satisfaction with their child’s body and the sex of the child. However, this variable was related to both male body satisfaction (χ²_(4)_ = 12.85, *p* < 0.01) and female body satisfaction (χ²_(4)_ = 22.56, *p* < 0.001).

**Table 1:** Body dissatisfaction in boys and girls.

	Boys ( *n *= 51) *n* (%)	Girls ( *n *= 60) *n *(%)	χ²_(1)_
**Children**			
Body satisfaction	23 (45.1)	25 (41.7)	
Body dissatisfaction	28 (54.9)	35 (58.3)	0.15
**Mothers**			
Child body satisfaction	23 (45.1)	34 (56.7)	
Child body dissatisfaction	28 (54.9)	26 (43.3)	1.60

Table 2 presents the percentages of child and maternal body satisfaction with their child’s bodies according to BMI. There was a significant association between body satisfaction and BMI in girls (χ²_(1)_ = 12.50, *p* < 0.001) and boys (χ²_(1)_ = 6.22, *p* < 0.05). These findings suggest that a greater BMI is related to body dissatisfaction for both sexes. Concerning maternal satisfaction, there was a significant association between maternal satisfaction and BMI for girls (χ²_(1)_ = 10.61, *p* < 0.005) but not for boys (χ²_(1)_ = 1.45, *p* = 0.34).

**Table 2:** Body dissatisfaction in boys and girls according to BMI.

	Boys ( *n* = 51)		χ²_(1)_	Girls ( *n* = 60)		χ²_(1)_
	Normal Weight *n* (%)	Overweight/Obese *n* (%)		Normal Weight *n* (%)	Overweight/Obese *n* (%)	
**Children**						
Body satisfaction	21 (93.3)	2 (8.7)		24 (96.0)	1 (4.0)	
Body dissatisfaction	17 (60.7)	11 (39.3)	6.22	19 (54.3)	16 (45.6)	12.50
**Mothers**						
Child body satisfaction	19 (82.6)	4 (17.4)		30 (88.2)	4 (11.8)	
Child body dissatisfaction	19 (69.9)	9 (32.1)	1.45	13 (50.0)	13 (50.0)	10.61

### 3.5. Eating Disturbance Predictors in Children

A regression analysis was conducted to predict child eating behavior using the global score of the ChEAT. Variables were entered in the following order: (i) child BMI; (ii) SPPC global self-esteem; (iii) child body dissatisfaction using CFD scores; (iv) maternal BMI; (v) maternal eating behaviors using the EDE-Q global score; and (v) maternal dissatisfaction with their child’s body using CFD scores. Two regression models were built to predict the eating behaviors of boys and girls separately. These models did not reveal multicollinearity problems, and the data were normally distributed [36].

The first regression controlled boys’ BMIs in the first block and found that this variable resulted in a significant model that accounted for 6% of the variance. Participants with higher BMIs reported more disordered eating behaviors. The self-esteem regression coefficients were not significant in the second block. We controlled body dissatisfaction in the third block and found that it resulted in a significant model that accounted for 14% of the variance. Participants with higher body dissatisfaction scores reported more disordered eating behaviors. The inclusion of the maternal dimensions in blocks four (BMI), five (EDE-Q), and six (satisfaction with child’s body) did not result in a significant model (see Table 3).

**Table 3:** Regression models that predict ChEAT global scores for boys and girls.

**Boys**					
	***R*^2^ (Adj. *R*^2^)**	***F***		**β**	***t***
**Block 1**—BMI	0.08 (0.06)	(1, 48) = 4.12		0.28	2.03 *
**Block 2**—Global self-esteem	0.08 (0.04)	(2, 47) = 2.03		0.02	0.13
**Block 3**—Body dissatisfaction	0.19 (0.14)	(3, 46) = 3.56		0.37	2.48 *
**Block 4**—Maternal BMI	0.20 (0.13)	(4, 45) = 2.77		0.10	−0.73
**Block 5**—Maternal EDE-Q global score	0.20 (0.11)	(5, 44) = 2.17		0.00	0.01
**Block 6**—Maternal dissatisfaction with child body image	0.22 (0.11)	(6, 43) = 1.18		0.15	1.01
**Girls**					
	***R*^2^ (Adj. *R*^2^)**	***F***	**β**		***t***
**Block 1**—BMI	0.05 (0.04)	(1, 58) = 3.11	0.23		1.77 ^+^
**Block 2**—Global self-esteem	0.19 (0.16)	(2, 57) = 6.66	−0.38		−3.12 **
**Block 3**—Body dissatisfaction	0.21 (0.16)	(3, 56) = 4.87	0.14		1.11
**Block 4**—Maternal BMI	0.34 (0.30)	(4, 55) = 7.18	0.38		3.38 **
**Block 5**—Maternal EDE-Q global score	0.41 (0.35)	(5, 54) = 7.41	−0.28		−2.41 *
**Block 6**—Maternal dissatisfaction with child body image	0.41 (0.34)	(6, 53) = 6.15	0.06		0.55

^+^ *p* < 0.10; * *p *< 0.05; ** *p *< 0.01.

The second regression controlled the girls’ BMI in the first block and found that it resulted in a marginally significant model that accounted for 4% of the variance. The second block added self-esteem, and this model explained 16% of the variance. Girls with low self-esteem reported more disordered eating behaviors. Block three added body dissatisfaction, and the model was not significant. The fourth block added maternal BMI, and it resulted in a significant model that explained 30% of the variance. Higher maternal BMIs predicted disordered eating behaviors in children. The inclusion of eating behavior in the fifth block resulted in a significant model that accounted for 35% of the variance. Maternal disordered eating behaviors predicted child disordered eating behaviors. Finally, maternal satisfaction with their child’s body was not a significant predictor of child eating disturbances in block six (see Table 3).

## 4. Discussion

This study investigated the differences between the eating behaviors and body satisfaction scores of preadolescent boys and girls as well as their mothers’ perceptions of these domains. This study also evaluated the predictors of eating problems using child BMI, self-esteem, and body satisfaction as well as maternal BMI, eating problems, and satisfaction with their child’s body.

No relationship was found between maternal BMI and either daughter or son BMI. This result is contrary to studies [15] showing that maternal BMI positively predicts overweight in daughters and those showing that parental BMI predicts child BMI [11].

As in previous studies [37,38], we did not find differences in the mean ChEAT scores of boys and girls. In this sample, 9.8% of boys and 8.3% of girls scored above the ChEAT screening threshold, whereas other studies found that 5% to 8% of boys and 9% to 14% of girls did so [38,39]. Gender differences in dieting and diet-related behaviors as assessed by the ChEAT and similar instruments are not usually apparent until children are 10 years old [3]. These findings are also consistent with maternal perceptions of their child’s eating behavior.

More than half of the sample reported body dissatisfaction, which is consistent with other studies [40]. In addition, this study highlighted the fact that boys are not immune to weight and body image concerns, a finding that has been reported in other studies [2]. In fact, approximately 55% of the boys reported body dissatisfaction, with 78.6% of those reporting the desire for a thinner body. This result was unexpected considering that previous research shows that boys frequently want to be bigger, whereas girls typically want to be thinner [5]. However, some studies have also found that boys want to be thinner. For example, Gustafson-Larson and Terry found that 45% of boys wanted to change their weight, with 38% wanting to be thinner and 7% wanting to be heavier [41]. Likewise, our results match previous research findings that boys younger than 11 years old are apparently not concerned about building muscles [42]. According to these authors, these results must be considered in light of the possible problematic use of body drawings to evaluate the body images of boys [5]. This unidimensional measure does not distinguish increased size due to fat from that due to muscle, and the selection of a desired body does not fully reveal the magnitude of the difference between real and ideal bodies. In addition, we did not find a relationship between sex and body satisfaction, which is contrary to many studies [5] that indicate there is a higher prevalence of body dissatisfaction among girls compared with boys. On the other hand, we found that greater BMI was related to higher body dissatisfaction for both sexes. Other studies [43] have also found a relationship between BMI and body dissatisfaction, with small but significant correlations between BMI and body dissatisfaction in 5 to 9-year-olds. 

Finally, the predictors of child eating disturbances differed by sex. Also, BMI and body dissatisfaction predicted eating disturbances in boys. Several studies have found that body dissatisfaction is related to weight concerns and eating problems in children and preadolescents [3]. Likewise, a greater BMI seems to be related to eating problems. BMI did not predict body dissatisfaction in girls. These results indicate that eating problems are related to BMI in boys, but they are softened related to BMI in girls. However, the literature consistently shows that both boys and girls with higher BMIs report more frequent diet and diet-related behaviors [18,38]. According to our findings, self-esteem predicted eating disturbances in girls but not in boys. Previous research has also shown that self-concept is associated with dieting concerns among children, especially for girls, and that the same relationship was weak and generally insignificant for boys [3].

This research found support for the influence of maternal BMI and eating behaviors on the eating disturbances of their daughters. Our results are consistent with previous findings [44] showing that mothers influence their daughters’ eating behaviors. Previous research suggests that maternal dieting predicts dieting concerns and behaviors in girls. Moreover, girls who reported that their mothers were on a diet were also most likely to demonstrate eating problems [3]. In contrast with these findings, our results failed to show a significant relationship between maternal eating behaviors and their sons’ eating behaviors. Therefore, boys appear to be less affected by maternal eating behaviors than girls [45]. This could result from differential parenting but also could be explained by biological factors. To our knowledge, this study is the first to separately evaluate eating disturbances in boys and girls with regard to the influence of both personal and maternal variables. Our findings highlight the possibility that maternal variables related to eating behaviors affect eating disturbances in girls more than in boys.

Some possible limitations of this research should be noted. First, the Cronbach’s alphas of the ChEAT in our sample are low. This finding suggests that this scale is not as reliable with younger children as it is with adults and older children [23]. As such, interpretations of these data should be cautious. Furthermore, this sample was limited to mothers and did not include fathers. More research is needed to understand the influence of fathers on child eating disturbances. Also, the convenient nature of the sample and the number of the participants included did not allow the generalization of the results. This aspect is important because 15% of obese children were found in the sample, which is higher than reported in other studies [46]. 

The retrospective, cross-sectional nature of our adopted design and its reliance on self-report questionnaires are also limitations. A large number of predictors compared to the sample size were used, and this constitutes a limitation to statistical power. Replication using larger samples and longitudinal designs are required to determine the predictors of eating disturbances in boys and girls. Future research addressing children’s eating behaviors and body satisfaction related to parental variables will be useful to the understanding of the developmental trajectory of children’s eating problems and body concerns. 

## 5. Conclusions

Boys and girls did not differ with regard to eating problems or body satisfaction. Child BMI and body image dissatisfaction predicted eating disturbances in boys, and self-esteem, maternal BMI, and eating behaviors predicted them in girls. These findings suggest that the maternal influences on child eating disturbances differ by sex.
